# Longer duration of operative time enhances healing metabolites and improves patient outcome after Achilles tendon rupture surgery

**DOI:** 10.1007/s00167-017-4606-7

**Published:** 2017-06-21

**Authors:** Simon Svedman, Olof Westin, Susanna Aufwerber, Gunnar Edman, Katarina Nilsson-Helander, Michael R. Carmont, Jón Karlsson, Paul W. Ackermann

**Affiliations:** 1 Integrative Orthopedic Laboratory, Department of Molecular Medicine and Surgery, Karolinska Institutet, Stockholm, Sweden; 2 Department of Orhopaedics, Institute of Clinical Sciences at Sahlgrenska Acadamy, University of Gothenburg, Gothenburg, Sweden; 3 Sahlgrenska University Hospital, Mölndal, Sweden; 4 Department of Psychiatry, Tiohundra AB, Norrtälje, Sweden; 5 Hallands Sjukhus, Kungsbacka, Sweden; 6 Department of Orthopaedic Surgery, The Princess Royal Hospital, Telford, Shropshire UK; 7 Department of Orthopedic Surgery, Karolinska University Hospital, Stockholm, Sweden

**Keywords:** Achilles tendon, Rupture, Operative time, Patient-reported outcomes, Physical activity, Pain, Post-operative complications, Microdialysis, Glutamate, Glycerol

## Abstract

**Purpose:**

The relationship between the duration of operative time (DOT), healing response and patient outcome has not been previously investigated. An enhanced healing response related to DOT may potentiate repair processes, especially in hypovascular and sparsely metabolized musculoskeletal tissues such as tendons. This study aimed to investigate the association between DOT and the metabolic healing response, patient-reported outcome and the rate of post-operative complications after acute Achilles tendon injury.

**Methods:**

Observational cohort, cross-sectional study with observers blinded to patient grouping. A total of two-hundred and fifty-six prospectively randomized patients (210 men, 46 women; mean age 41 years) with an acute total Achilles tendon rupture all operated on with uniform anaesthetic and surgical technique were retrospectively assessed. At 2 weeks post-operatively, six metabolites were quantified using microdialysis. At 3, 6 and 12 months, patient-reported pain, walking ability and physical activity were examined using self-reported questionnaires, Achilles tendon total rupture score, foot and ankle outcome score and physical activity scale. At 12 months, functional outcome was assessed using the heel-rise test. Complications, such as deep venous thrombosis, infections and re-operations, were recorded throughout the study.

**Results:**

Patients who underwent longer DOT exhibited higher levels of glutamate (*p* = 0.026) and glycerol (*p* = 0.023) at 2 weeks. At the 1-year follow-up, longer DOT was associated with significantly less loss in physical activity (*p* = 0.003), less pain (*p* = 0.009), less walking limitations (*p* = 0.022) and better functional outcome (*p* = 0.014). DOT did not significantly correlate with the rate of adverse events, such as deep venous thrombosis, infections or re-ruptures. Higher glutamate levels were associated with less loss in physical activity (*p* = 0.017). All correlations were confirmed by multiple linear regressions taking confounding factors into consideration.

**Conclusion:**

The results from this study suggest a previously unknown mechanism, increased metabolic response associated with longer DOT, which may improve patient outcome after Achilles tendon rupture surgery. Allowing for a higher amount of traumatized tissue, as reflected by up-regulation of glycerol in patients with longer DOT, may prove to be an important surgical tip for stimulation of repair of hypometabolic soft tissue injuries, such as Achilles tendon ruptures.

**Level of evidence:**

II.

**Electronic supplementary material:**

The online version of this article (doi:10.1007/s00167-017-4606-7) contains supplementary material, which is available to authorized users.

## Introduction

Duration of operative time (DOT), i.e. knife time, can be highly variable and be associated with conflicting outcomes. Longer DOT has been shown, for example, in bariatric surgery, to be related to increased complication rate of surgical site infections and deep venous thrombosis [1, 2, 6, 19, 33]. In hernia surgery, on the other hand, longer DOT was correlated with less risk of re-operation [35]. Whether DOT can affect the metabolic healing response from surgical repair after tissue injury has not previously been investigated.

Conceivably, prolonged DOT may be associated with more surgical tissue wounding, resulting in an increased cellular metabolism, which may enhance tissue repair, especially in hypovascular and sparsely metabolized musculoskeletal tissues, such as tendons. However, DOT has not been compared to outcome in relation to tendon surgery.

Repair after acute Achilles tendon rupture (ATR) is associated with an up-regulation of essential metabolites, glycerol, glutamate, glucose, lactate and pyruvate, all involved in the healing process [13]. Specifically glycerol, a marker of cellular damage [20], and glutamate, a metabolite/neurotransmitter, can promote wound healing [5, 22], yet the effect of DOT on these metabolites is not known.

Healing outcome after ATR is very variable with many patients exhibiting loss of physical activity level, pain and walking deficits [7, 11, 15, 17, 24, 26, 36]. The prolonged healing time and variable outcome after surgical repair of ATR have been attributed to an underlying degenerative pathology (tendinopathy) [3, 16] and a low metabolic rate [13], which may be influenced by a longer DOT and elevated levels of metabolites [13, 34].

In the present study, it was hypothesized that DOT may affect metabolic healing response and, thus, influence patient outcome after surgical repair of ATR. The aim of the study was to assess DOT in relation to early metabolic healing, 3-, 6- and 12-month patient-reported outcome of pain and walking deficits as well as functional outcome at 1 year. Moreover, adverse events and complications such as deep venous thrombosis, infections and re-operations were also assessed in relation to DOT in regard to ATR surgery.

## Materials and methods

### Patients

**Fig. 1:**
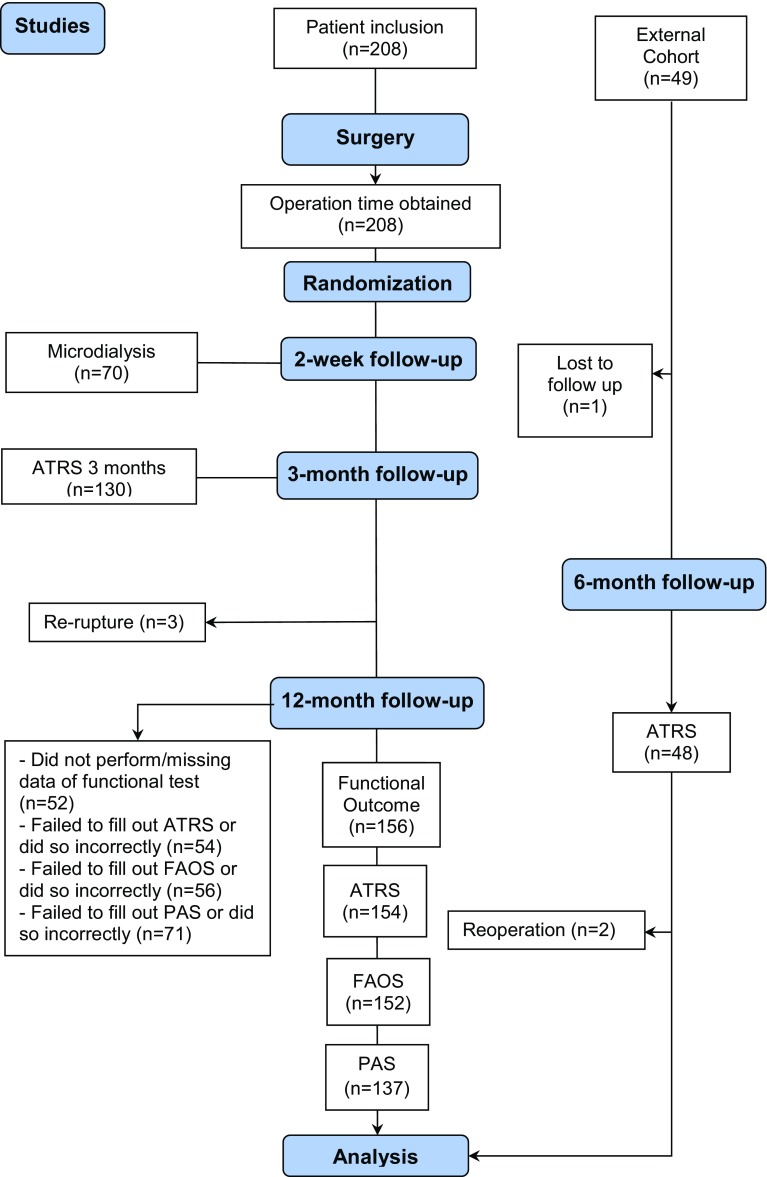
Follow-up flowchart showing patient inclusion and follow-up. *ATRS* Achilles tendon total rupture score, *FAOS* foot and ankle outcome score, *PAS* physical activity scale

Two-hundred and eight patients were included from three different randomized control trials at Karolinska University Hospital, Stockholm, Sweden. Patient inclusion and follow-up are described (Fig. 1).

Patients, who had sustained an acute unilateral rupture of the Achilles tendon, were eligible for inclusion. Exclusion criteria were: current anticoagulation treatment (including high-dose acetylsalicylic acid), known kidney failure, heart failure with pitting oedema, thrombophlebitis, thromboembolic event during the previous 3 months, known malignancy, haemophilia, pregnancy, other surgery during the previous month, inability to follow instructions or planned follow-up at another hospital. All patients were operated on at the Karolinska University Hospital, Stockholm, by the surgeon responsible for outpatient surgeries that day. Accordingly, the patients could not request a specific surgeon. Patients were enrolled and assigned to the post-operative interventions by a research nurse or a third-party nurse. Randomization to post-operative treatment was performed using computer-generated random numbers in permuted blocks of four, through an independent software specialist, and consecutively, numbered, sealed, opaque envelopes opened after surgery and prior to treatment. At 2 weeks post-operatively, microdialysis was performed on the Achilles tendon of both limbs of 70 patients, who consented to undergo microdialysis. At a 3-month follow-up, 130 patients filled out the Achilles tendon total rupture score (ATRS) [27]. Additionally, at the 12-month follow-up, 156 patients filled out the ATRS-, foot and ankle outcome score-(FAOS) and physical activity scale (PAS) survey and patients were assessed for functional heel-rise (Fig. 1).

#### Controlling external cohort

To confirm the data from the internal cohort of 208 patients, ATRS data at 6 months post-operatively from an external cohort of 48 ATR patients from Sahlgrenska University Hospital in Gothenburg, Sweden, were included in the analyses. These patients were part of a randomized controlled trial comparing surgical and non-surgical treatment with early mobilization [26], using a modified Kessler suture technique. Anaesthetic methods varied between local, spinal and general anaesthesia.

### Patient characteristics

Described in Table 1.

**Table 1:** Patient demographics

Variable	Internal cohort	External cohort
Gender [%, (n)]		
Male	81 (169)	85 (41)
Female	19 (39)	15 (7)
Total	100 (208)	100 (48)
Age, mean ± SD (years)	40 ± 8	41 ± 9
Length, mean ± SD (cm)	178 ± 8	179 ± 9
Weight, mean ± SD (kg)	84 ± 13	86 ± 14
BMI, mean ± SD (kg/cm^2^)	26 ± 3	27 ± 3
Smoker [%, (n)]		
No	93 (187)	–
Yes	7 (14)	–
Op. time mean ± SD (min)	37 ± 13	40 ± 12
Complications [%, (n)]		
DVT occurrence	41 (84)	29 (14)
Re-op. (re-rupture)	2 (3)	2 (1)
Infections	2 (3)	4 (2)

*SD* standard deviation, *cm* centimetres, *kg* kilogram, *BMI* body mass index, *Op. time* duration of operative time, *DVT* deep venous thrombosis, *Re*-*op*. re-operations

### Duration of operative time (DOT), patient characteristics and treatment

Out of the patient characteristics and treatment factors (gender, age, body height, weight and BMI, and treatment group), only patient height was significantly and positively associated with longer DOT (*r* = 0.204, *p* = 0.004).

### Surgical procedure

All patients enrolled were operated according to a standardized technique (see Additional file 1) [9]. The patient was placed in prone position, and thereafter, local anaesthetic was introduced to the skin, subcutis and peritendinous space. A medial incision was made over the Achilles tendon followed by a central incision, through fascia cruris and the paratenon. A modified Kessler suture technique using two 1–0 polydioxanone was used in bringing the tendons together. The paratenon and fascia cruris was closed using a 3–0 vicryl suture and the skin with 3–0 ethilon suture. The DOT was recorded and defined as minutes from commencement of skin incision to completion of skin closure.

### Additional file 1: operating regimen for Achilles tendon surgery (Mp4)

The video demonstrates the standardized operating regimen used in all patients. After dissecting through the skin, fascia cruris and paratenon, the tendon stumps were sutured together by a modified Kessler technique using two polydioxanone sutures.

### Post-operative treatment

The post-operative regimens varied mainly during the first two post-operative weeks. Seventy-five patients were treated with calf intermittent pneumatic compression (IPC) under a brace [8]; forty-four patients were treated functional weight-bearing (FWB) [34]; and eighty-nine patients were treated in a plaster cast immobilization (IMM) [34]. The external cohort patients were all treated in plaster cast (IMM) [26].

#### Mobilized and full weight-bearing group (FWB)

The patients randomized for orthosis treatment initiated functional mobilization directly post-operatively with the VACO^®^ped (OPED GmbH, Germany), with adjustable range of motion of the ankle. During the first two post-operative weeks, 15°–30° of plantar flexion was allowed. At 2 weeks post-operatively, the range of motion was increased to 5°–30° of plantar flexion for the remaining 4 weeks. Full weight-bearing with crutches and range of motion exercises were allowed after application of the orthosis. 1-h daily non-weight-bearing range of motion exercises without the orthosis was recommended.

#### Immobilized and non-weight-bearing group (IMM)

The IMM groups received a conventional non-weight-bearing below-knee plaster cast with the ankle in 30° of equinus position. At 2 weeks post-operatively, the cast was replaced with a removable walker (Aircast^®^ Standard walking brace) with three heel wedges for the remaining 4 weeks of immobilization. Every consecutive week, one heel wedge was removed. Full weight-bearing with crutches was allowed after application of the orthosis. One-hour daily non-weight-bearing range of motion exercises without the orthosis was recommended.

### Microdialysis

To assess tendon healing at 2 weeks, microdialysis followed by metabolite quantification of glutamate, glycerol, glucose, lactate, pyruvate and lactate-to-pyruvate ratio was performed on patients willing to undergo this procedure (*n* = 70), using the method as described earlier [21]. The patients were placed in prone position, and the skin covering the Achilles tendons was sterilized bilaterally. The calcaneus was identified, and the microdialysis catheter (CMA 71; CMA Microdialysis AB, Solna, Sweden; 100 kDa molecular cut-off membrane, 0.5 mm outer diameter; 30 mm in length) was inserted 1.5–3 cm proximal of the calcaneus and 1 cm laterally of the Achilles tendon. Using ultrasound guidance, the catheter membrane was inserted as close to the site of rupture as possible in the peritendinous space and in the same location on the contralateral tendon. After insertion, Macrodex^®^ perfusion fluid was pumped through the catheter, via the tip of the catheter and the semi-permeable catheter membrane, into a vial. Four vials were collected from each tendon, with a flowrate of 1.0 µL/min and 2 vials/h, and analysed within five days using an ISCUS Clinical Microdialysis Analyzer. The first vial was discarded consistently since the insertion of the catheter might alter the concentrations of the substances at the tendon. The other vials were used to calculate individual and overall mean concentrations of individual substances or ratios, which were used for statistical analyzation.

### Patient-reported outcome measures (PROMs)

Each patient answered questionnaires (ATRS, Swedish, version 6; FAOS, Swedish, version LK 1.0; PAS) at the 3-, 6- (external cohort) and 12-month follow-up appointment in order to determine the patient’s degree of symptoms. The ATRS includes specific questions (scored from 0 to 10, 10 indicating no limitations) regarding pain (Q4: Are you limited due to pain in your calf/Achilles tendon/foot?) and walking (Q6: Are you limited when walking on uneven surfaces?), which were considered the two most important PROMs in this study [27]. The FAOS uses five categories of questions (pain, quality of life, sports and recreation, symptoms and activity of daily living), with a maximum score of 100 in each category, 100 indicating no limitation [30]. For the PAS, a score of 1 indicates that a patient is mostly sedentary, whereas a score of 6 means that a patient has engaged in heavy physical exercise several times per week [28].

### Functional outcome

Patients were evaluated with regard to their foot and Achilles tendon function, using an established validated regimen of the heel-rise endurance test, at the 12-month follow-up [21, 25, 32].

### Statistical analysis

All data were entered in SPSS version 22 (IBM SPSS, version 22.0. Armonk, NY, USA). The variables were summarized with standard descriptive statistics such as mean, standard deviation and frequency. All variables were checked for severe skewness. Comparisons between groups were performed using ANOVA for repeated measurements (Group × Time) and independent Student’s *t* test when appropriate. Correlations between different variables and outcome were expressed as Pearson’s correlations coefficients. Nonparametric Spearman’s rank correlation was used if a distribution was severely skewed. For outcome variables that were normally distributed and significantly correlated with DOT, multiple linear regressions analyses (stepwise forward method, with an inclusion level of 0.05) were conducted. This was done in order to investigate the unique relationships between the independent variables (gender, age, length, weight, BMI, surgeon experience, the time from injury until operation, DOT and post-operative treatment) and the dependent variable. The level of significance was ≤5% for all analyses.

## Results

### Outcome at 2 weeks

#### DOT and healing metabolites

Longer DOT correlated significantly with increased levels of glycerol (*p* = 0.023) and glutamate (*p* = 0.026), when controlling for potential confounding factors (Table 2). The levels of glutamate and glycerol exhibited no relationship (n.s.). The other metabolites were not significantly related to DOT (Table 3).

**Table 2:** Duration of operative time (DOT) is the strongest variable for patient outcome

Dependent outcome variable	Mean value (SD)	Patients with residual symptoms % (*n*/*N*)*	Univariate correlations	Multiple linear regression result
12 months				
Loss in physical activity (PAS)	0.75 (1.0)	61 (84/137)	*r* = −0.253, *p* = 0.003	*F*(1, 132) = 9.217, *p* = 0.003, *R* ^2^ = 0.065
Pain (FAOS)	94.6 (8.8)	43 (65/152)	*r* = 0.194, *p* = 0.017	*F*(1, 147) = 5.723, *p* = 0.018, *R* ^2^ = 0.037
Pain (ATRS)	8.8 (1.9)	44 (68/154)	*r* = 0.210, *p* = 0.009	*F*(1, 149) = 7.088, *p* = 0.009, *R* ^2^ = 0.045
Heel-rise repetitions ratio (injured/uninjured)	0.8 (0.2)	37 (57/155)	*r* = 0.197, *p* = 0.014	*F*(1, 147) = 5.723, *p* = 0.018, *R* ^2^ = 0.037
Walking limitations (ATRS)	8.8 (1.8)	48 (74/153)	*r* = 0.185, *p* = 0.022	*F*(1, 148) = 5.333, *p* = 0.022, *R* ^2^ = 0.035
6 months				
Walking limitations (ATRS)	8.5 (1.5)	70 (33/47)	*r* = 0.327, *p* = 0.025	*F*(1, 45) = 4.495, *p* = 0.025, *R* ^2^ = 0.107
3 months				
Pain (ATRS)	7.0 (2.6)	80 (105/130)	*r* = 0.176, *p* = 0.045	*F*(2, 126) = 4.848, *p* = 0.009, *R* ^2^ = 0.071
2 weeks				
Glycerol levels	89.9 (86.1)	N/A	*r* = 0.281, *p* = 0.023	
Glutamate levels	82.0 (31.7)	N/A	*r* = 0.308, *p* = 0.026	*F*(1, 48) = 5.904, *p* = 0.019, *R* ^2^ = 0.110

The independent variables considered in the multiple linear regression analysis were: gender, age, length, weight, BMI, surgeon experience, time to operation, duration of operative time (DOT) and post-operative treatment. DOT is the strongest independent variable for patient outcome in all analyses

*PAS* physical activity scale [1–6], *FAOS* foot and ankle outcome score [0–100], *ATRS* Achilles tendon total rupture score [0–10]

* Patients with residual symptoms were categorized as ≥1 loss in PAS, ≤99 in FAOS, ≤9 in ATRS and ≤0.8 in heel-rise ratio between injured and uninjured legs

**Table 3:** Descriptive data of outcome variables, dichotomized in short and long operative duration

Outcomes	≤34 min	>35 min	*P* value
	Mean (SD)	Mean (SD)	
2 weeks—metabolites			
Glucose	2.72 (0.76)	2.71 (0.74)	n.s.
Lactate	1.75 (0.73)	1.59 (0.89)	n.s.
Pyruvate	90.65 (24.89)	89.13 (29.33)	n.s.
Glycerol	62.88 (22.72)	107.84 (106.36)	**0.014**
Glutamate	78.35 (30.44)	84.50 (32.72)	n.s.
Lactate–pyruvate ratio	19.12 (5.14)	18.56 (6.56)	n.s.
3 months—patient-reported			
Pain (ATRS)	6.57 (3.03)	7.46 (2.02)	**0.048**
Walking limitations (ATRS)	5.09 (2.61)	5.63 (2.58)	n.s.
6 months—patient-reported*			
Pain (ATRS)	8.25 (1.75)	8.17 (2.25)	n.s.
Walking limitations (ATRS)	8.00 (1.69)	9.00 (1.09)	**0.021**
12 months—patient-reported			
Pain (ATRS)	8.51 (2.28)	9.18 (1.49)	**0.016**
Walking limitations (ATRS)	8.40 (2.03)	9.19 (1.35)	**0.006**
Pain (FAOS)	92.86 (10.37)	96.15 (6.88)	**0.025**
Change in physical activity level	1.05 (0.96)	0.49 (1.02)	**0.001**
12 months—functional			
Number of heel-rises—injured side	24.01 (8.82)	25.93 (7.80)	n.s.
Number of heel-rises—uninjured side	29.59 (8.97)	30.78 (8.23)	n.s.
Limb symmetry index—repetitions	0.83 (0.23)	0.86 (0.20)	n.s.

The outcome variables are dichotomized into two groups (short and long operative time) by the median operative time. Bold indicates a significant *p* value less than 0.05. The dichotomized data of the metabolite glutamate and the functional outcome are in contrast to the multiple regression analyses, not significantly different between the groups

*ATRS* Achilles tendon total rupture score, *FAOS* foot and ankle outcome score, *PAS* physical activity scale, *n.s.* non-significant

* Separate data from the external cohort and therefore dichotomized by separate median duration of operative time of <42 and ≥42 min

### Outcome at 3 and 6 months

#### DOT, pain and walking ability

Longer DOT was at 3 months post-surgery associated with less experience of pain (*p* = 0.045), but not significantly correlated with limitations in walking on uneven surfaces (Table 2).

Longer DOT was at 6 months associated with improved walking on uneven surfaces (*p* = 0.025), but not to the experience of pain (n.s.; Table 2).

### Outcome at 12 months

#### DOT, pain and walking ability

At 1 year, 44% of the patients experienced limitations due to pain in their limb. Longer DOT correlated with less experience of pain in both the ATRS (*p* = 0.009) and FAOS (*p* = 0.017) questionnaire, which was confirmed by the regression analysis (Table 2). Limitations in walking on uneven surface were found in 48% of the patients. Longer DOT showed a significant association with less limitation in walking on uneven surfaces (*p* = 0.022), corroborated by multiple linear regression (Table 2).

#### DOT and change in physical activity score (PAS)

**Fig. 2:**
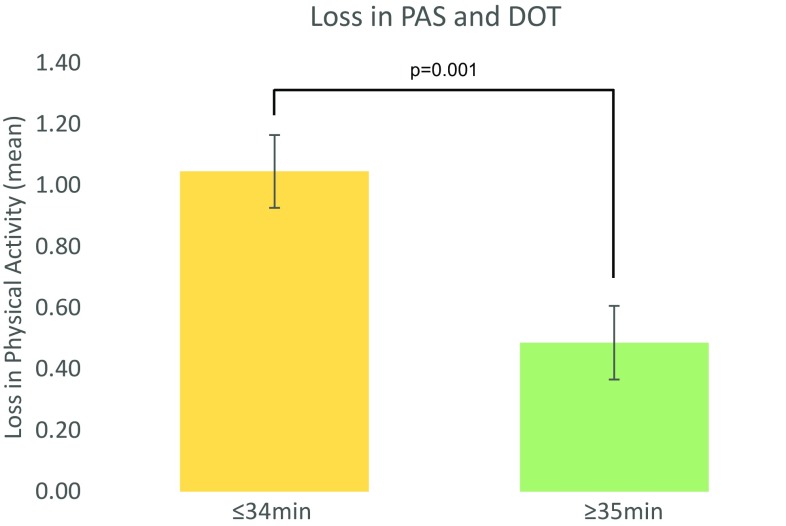
Loss in PAS and DOT. Column chart of duration of operation time (DOT) versus loss in physical activity (PAS) when dichotomized at median time of DOT (*n* = 137). Loss in PAS is calculated as the difference between PAS score pre-injury and PAS score at the 1-year follow-up

Longer DOT was significantly correlated with less loss of physical activity, i.e. difference in PAS pre- and 1 year post-operatively (*p* = 0.003), confirmed by multiple linear regression (Table 2). Patients with a short DOT (<35 min) exhibited a mean loss of 1.05 in PAS, while patients with long DOT (≥35 min) had a mean loss of 0.49 (*p* = 0.001; Table 3; Fig. 2).

Higher PAS at 12 months was associated with less experience of pain (*r* = 0.197, *p* = 0.019) and fewer limitations in walking on uneven surfaces (*r* = 0.236, *p* = 0.005). Multiple linear regression confirmed less limitations in walking on uneven surfaces as the most important factor for increased PAS at 12 months (*r* = 0.231, *p* = 0.006).

#### DOT, metabolites and PAS

The relationship between metabolite levels and outcome at 12 months was examined. Higher concentration of glutamate was associated with less loss in physical activity (*r* = − 0.426, *p* = 0.017). Multiple linear regression confirmed this relationship. The levels of glycerol were not associated with any of the outcome variables examined at 12 months.

#### DOT and functional outcome

Longer DOT was significantly associated with improved limb symmetry index of repetitions (*p* = 0.014; Table 2), but not with concentric heel-rise power, total power or heel-rise height (n.s.).

### Professional experience of the surgeon

General professional experience of the surgeon (resident = 0 or specialist = 1; *r* = −0.524, *p* < 0.001) and specific professional experience (*r* = −0.192, *p* = 0.007), i.e. the number of ATR operations performed by an individual surgeon in the study, were related to significantly shorter DOT. However, when multiple linear regression was performed with outcome as dependent variable and the surgeons general and ATR-specific experience was included as independent variables together with DOT, only DOT was significant.

### Adverse events

Longer DOT was not associated with higher occurrence of re-ruptures (n.s.), deep venous thrombosis (n.s.) or infections (n.s.). Review of the operative reports demonstrated consistent operative techniques compliant with the predefined surgical protocol. Hence, surgeons dichotomized in short and long DOT exhibited no differences in compliance with the predefined surgical protocol (n.s.).

## Discussion

This study demonstrated that longer DOT was significantly associated with up-regulation of metabolites essential for healing and less loss in physical activity at 12 months post-operatively. Moreover, longer DOT was related to less patient experience of pain and increased ability to walk on uneven surfaces. This study showed no relationship between adverse events and complications related to DOT with ATR surgery. This is the first study to establish a relationship between duration of operative time and patient outcome including healing response after a musculoskeletal tissue injury.

The most important finding of the present study was to establish less loss in physical activity in the patients that underwent longer DOT. This result reflected a substantial difference in activity level when dichotomizing the patient into short (≤34 min) or long DOT (>35 min). Patients with long DOT only experienced half of the reduction in the physical activity scale as compared to the patients with short DOT, i.e. one step. One-step loss on the PAS survey, e.g. from 4 to 3, equals to only being able to perform light physical activity, i.e. walking or gardening 1–2 h per week, instead of hard physical activity, i.e. running and tennis for the same amount of time. Such reductions in patient-reported outcome are clinically significant and may potentially be associated with adverse health-related effects [10].

The finding of longer DOT being associated with less loss in physical activity may be accounted for by the patients experiencing less limitations of pain and difficulty of walking on uneven surfaces. With less symptoms, patients would be expected to be more active and so suffer a reduced decline in activity and a quicker return to an active lifestyle. Furthermore, less pain and less walking limitations also correlated with higher physical activity levels. Since almost half of the patients experienced pain and walking limitations at 1 year post-operatively, this suggests these are not the only factors and further identification of underlying factors on how DOT affects pain, walking and physical activity remains vital for optimizing patient outcome.

The findings of the present study that longer DOT appeared to improve both functional and patient-reported outcome are novel. Earlier research on DOT, though in different types of surgery, have mostly reported negative impact of longer DOT with greater risk of complication, prolonged length of hospital stay and deep venous thrombosis or pulmonary embolism in, for example, bariatric surgery [2, 4, 29]. This study did not support any differences in complication rates, i.e. occurrence of deep venous thrombosis and infections, related to DOT.

In earlier studies on patients receiving herniorrhaphy, longer DOT resulted in fewer re-operations, due to the avoidance of intra-operative technical failures [35]. In the present study, DOT did not significantly affect the risk of re-operation due to re-ruptures. A review of the operative reports in question verified that neither long nor short DOT was associated with increased risk of complications. Therefore, it is concluded that longer DOT was not primarily associated with less major technical failures during ATR surgery.

To control for potential procedural differences between surgeons, all surgical procedures were performed with uniform anaesthetic and operative techniques, which were defined in the study protocol [8]. Moreover, the operative reports demonstrated no significant differences in operative techniques used between surgeons with longer and shorter DOT.

The findings showing that broader experience and more procedural training of the surgeon were significantly associated with a shorter DOT imply that the improved outcome after longer DOT was not related to a higher either general or ATR-specific experience of the surgeon. This was confirmed by multiple linear regression demonstrating that DOT, rather than surgeon experience, was the determining variable for outcome.

The second main finding of this article pertains to the establishment of a positive association between longer DOT and increased levels of the metabolites glutamate and glycerol. These metabolic markers of repair progress were assessed at the 2-week follow-up by the use of microdialysis.

The elevated glycerol levels associated with longer DOT suggest increased wounding of the tissue related to DOT, since glycerol is a marker of cell damage [12, 20]. This conclusion is logical also in the context that the less experienced surgeons who exhibited extended DOT may need to have greater exposure and more tissue damage to perform the tendon repair. Increased cell damage and the observed elevation of metabolites in ATR patients with longer DOT may also suggest a potential general up-regulation of the synthesis of growth factors [23].

The increased concentration of glutamate in the tendons which underwent longer DOT strengthens the suggestion of an improved healing response, since glutamate has been found up-regulated during tendon repair and is implicated in enhancing the healing process [31, 34]. Glutamate contributes to wound healing by chemotaxis of neutrophils [14] and has in tendon healing been speculated to improve angiogenesis, cell proliferation and nerve ingrowth [31].

Interestingly, the observed elevated glutamate levels were significantly associated with an improved patient outcome, i.e. less loss in physical activity level. This finding further strengthens the concept that increased glutamate concentration promotes tendon healing [13, 34]. Higher glutamate levels, however, were not related to less pain or less walking difficulties, indicating that there may be other factors by which glutamate improves physical activity.

One potential limitation of this study is that the correlation coefficients presented could be categorized as weak according to general, simplified guidelines [18]. Such guidelines are mostly used for agreement between observer ratings for categorical data. As this was not a test–retest setting, but observation of the association of independent variables, it is argued that the results, showing significant associations, are not negligible and might be clinically significant. Another limitation might be that the patients of the different cohorts performed slightly different post-operative rehabilitation protocols. However, the rehabilitation protocols were taken into account in the statistical analyses and did not affect the outcome. Although the study was controlled for possible confounding factors, this was a cohort study with its potential bias. The results therefore warrant prospective randomized trials to corroborate the current findings.

## Conclusion

This study demonstrates that longer DOT is associated with improved patient-reported and functional outcome 1 year after surgery of an acute ATR. Longer DOT was associated with reduced loss of physical activity level, less pain and fewer walking limitations and without increased occurrence of adverse events.

The current data suggest a biological explanation. The increased levels of glycerol indicate that surgeons with longer DOT allowed more traumatized tissue, which may be an essential surgical trick to provoke better repair responses in hypometabolic and tendinopathic injuries such as ATR. The provoked response resulted in an up-regulation of glutamate, which is known for enhancement of healing processes.

This biological finding may change surgical practice and therefore calls for prospective trials, which examine novel operative and biological techniques to stimulate the repair of low metabolized tissues, such as the Achilles tendon.

## Electronic supplementary material

Below is the link to the electronic supplementary material.

Supplementary material 1 (MP4 294728 kb)
